# Tuberculous Trochanteric Bursitis Mimicking Hydatidosis: A Rare Case Report

**DOI:** 10.7759/cureus.94693

**Published:** 2025-10-16

**Authors:** Mouncef Amahtil, Amine Hamzaoui, Khalil Ouda, Sadougui Mohamed, Abdelkrim Daoudi

**Affiliations:** 1 Orthopedics and Traumatology, Mohammed VI University Hospital, Faculty of Medicine and Pharmacy of Oujda, Mohamed I University, Oujda, MAR

**Keywords:** case report, hydatidosis differential diagnosis, primary tuberculosis, tuberculous trochanteric bursitis, uncommon presentation

## Abstract

Tuberculous trochanteric bursitis is a rare condition that accounts for less than 2% of musculoskeletal tuberculosis cases, usually diagnosed late because of reduced diagnostic suspicion, and the insidious clinical presentation aspect of musculoskeletal tuberculosis. We describe the case of a 26-year-old male presenting with a slowly enlarging painful mass of the right hip without systemic symptoms. Imaging initially suggested hydatidosis, but aspiration confirmed Mycobacterium tuberculosis. A complete bursectomy combined with antitubercular therapy led to full recovery with no recurrence at two years. This case highlights the diagnostic challenges of tuberculous trochanteric bursitis, often mimicking other pathologies, and underlines the importance of early suspicion, histopathological confirmation, and combined surgical and medical treatment for optimal outcomes.

## Introduction

Extrapulmonary tuberculosis may involve multiple organs, producing a wide range of clinical presentations. Mycobacterium tuberculosis may invade soft tissues, extending into muscles, tendinous structures, fascial planes, bursae, and synovial membranes [1].

Primary tuberculous trochanteric bursitis (TTB), a relatively common manifestation of the disease before the advent of antituberculous therapy, is currently an exceptional disorder that constitutes less than 2% of osteoarticular tuberculosis manifestations [2]. The bursae most often affected are those prone to repeated trauma, such as the trochanteric, olecranon, and subdeltoid bursae [3].

The identification and management of tuberculous trochanteric bursitis may be postponed for several years because of the scarcity of signs, its slow clinical course, and a low index of suspicion. It is noteworthy that fever or systemic complaints are usually absent in patients with tuberculous trochanteric bursitis [4]. Consequently, it may be readily missed and ignored, leading to a delay in therapy and compromising the patient’s quality of life. In fact, the clinical expression of tuberculous trochanteric bursitis often lacks precision and may mimic tumors or other pathologies, including trochanteric bursitis of other etiologies (such as inflammatory or traumatic), soft tissue tumors (such as sarcoma or lipoma), septic bursitis, metastatic lesions, and chronic osteomyelitis [2].

The aim of this case report is to highlight the diagnostic difficulties of primary tuberculous trochanteric bursitis and to address the lack of recent literature describing its atypical presentation and delayed diagnosis.

## Case presentation

**Figure 1:**
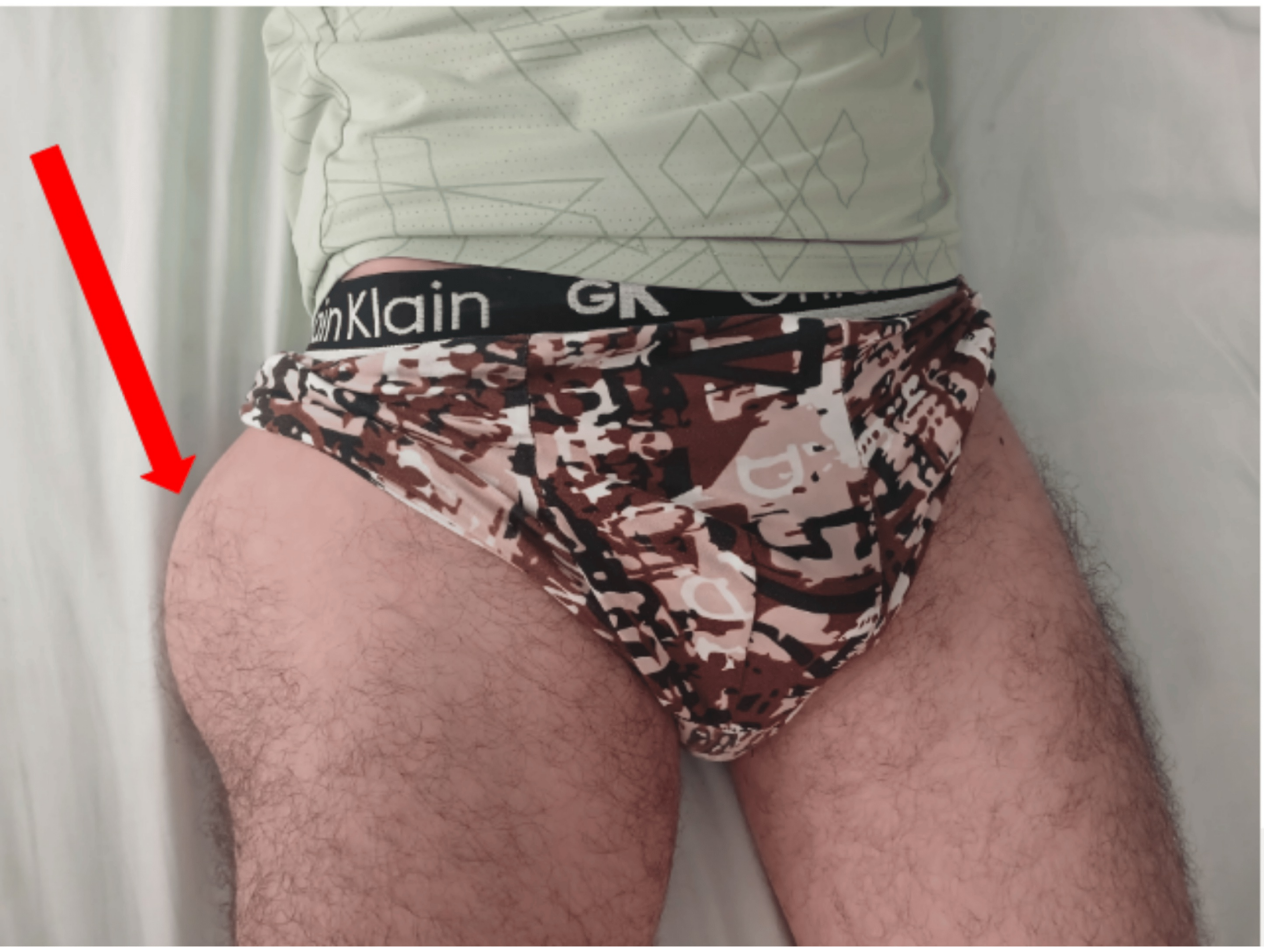
Clinical view of the mass located over the left greater trochanter (arrow).

**Figure 2:**
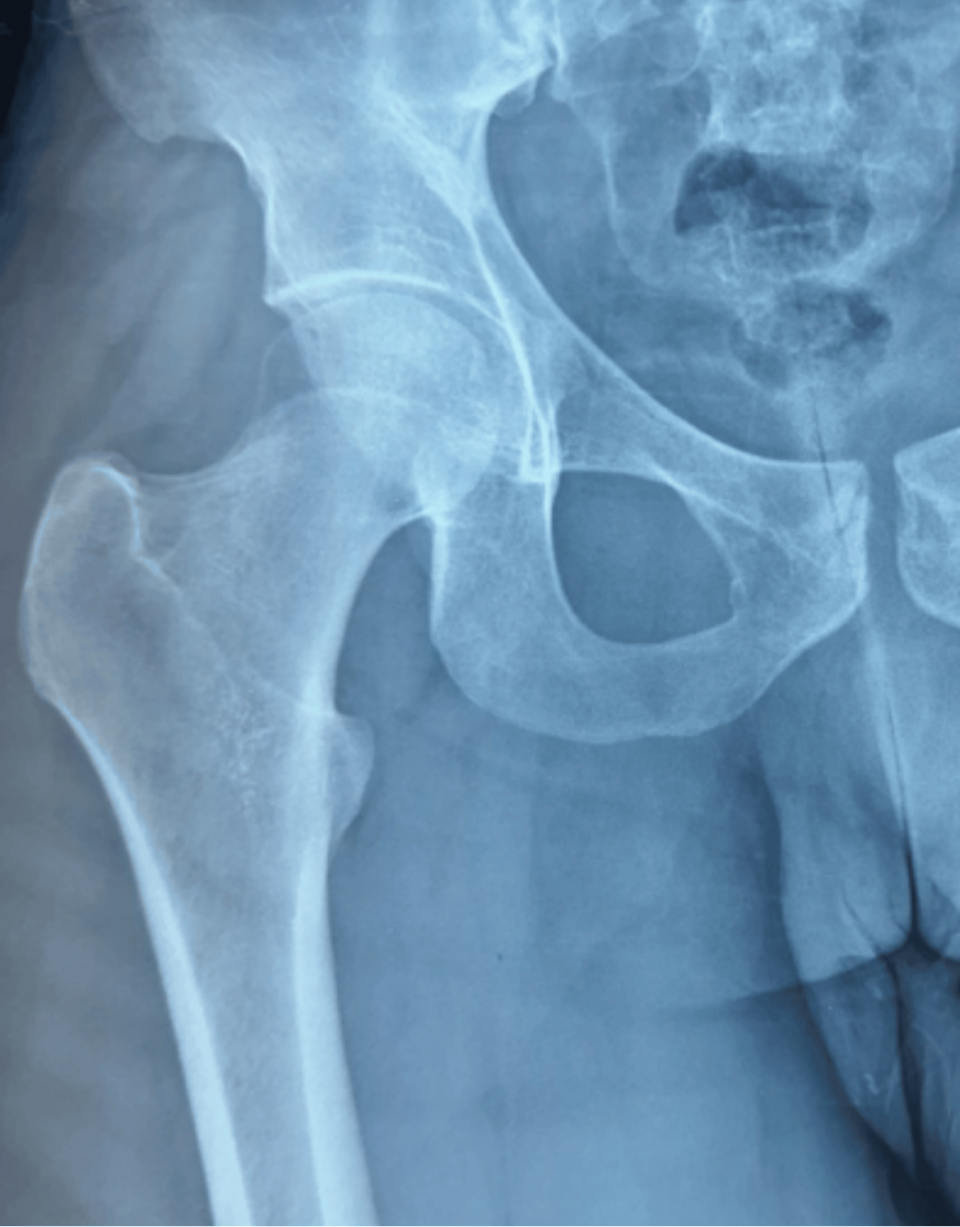
Normal standard right hip radiograph.

A 26-year-old male presented to the orthopedic department with right hip pain associated with a mass fixed to the lateral side of his right thigh. The mass was large but had been growing slowly over the last 12 months. The patient had no referred pain and was still walking. Fever, weight loss, and night sweats were denied. On examination, he had a 14-cm mass fixed to his pelvis on the right side (Figure 1). Pain was evoked by pressure and mobilization of the hip. Laboratory testing revealed a normal erythrocyte sedimentation rate within normal limits. All other standard laboratory investigations yielded normal results. A conventional chest X-ray revealed no pathological findings. Conventional radiography of the hip demonstrated soft-tissue enlargement in the left trochanteric area, without evidence of osseous lesions (Figure 2).

**Figure 3:**
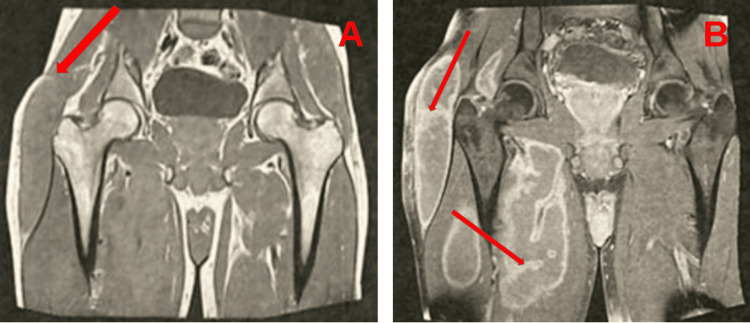
MRI of the left hip (A) Coronal non-contrast image demonstrating a collection arising from the trochanteric bursa (arrow). (B) Coronal contrast-enhanced image showing multiple enhancing parietal villosities (arrows), without signs of locoregional aggressiveness or involvement of adjacent musculotendinous and osseous structures, suggesting hydatidosis.

Ultrasound and magnetic resonance imaging of the hip revealed a bulky mass with impure fluid content showing multiple enhancing parietal villosities, without signs of locoregional aggressiveness or involvement of adjacent musculotendinous and osseous structures, suggesting hydatidosis (Figure 3). However, hydatid serology was negative. Ultrasound-guided aspiration of the abscess yielded a caseous-like material. Polymerase chain reaction (PCR) for Mycobacterium tuberculosis complex DNA was positive.

**Figure 4:**
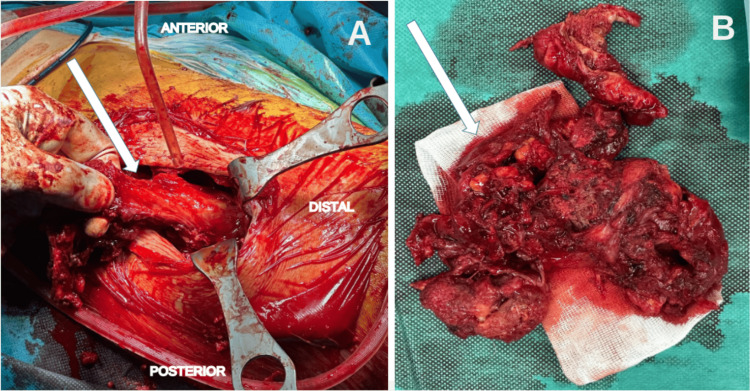
Intraoperative findings (A) Excision of the mass originating from the greater trochanter (arrows). (B) Appearance of the mass after resection.

**Figure 5:**
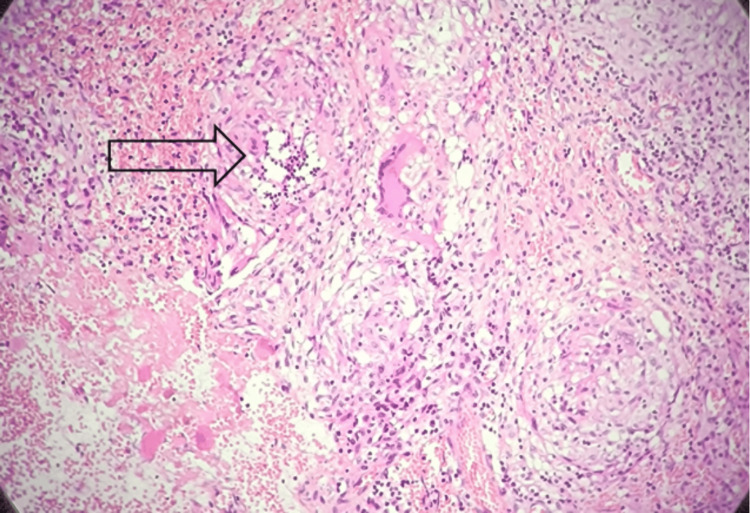
Microscopic examination of the mass biopsy (Hematoxylin and Eosin staining) showing multiple epithelioid and multinucleated giant cell granulomas of varying size, composed of epithelioid cells and giant cells, surrounded by a lymphocytic rim and centered by caseous necrosis (arrow).

Surgical treatment was chosen, and the patient underwent drainage of the collection. During the procedure, a whitish, pus-like fluid was observed. A complete bursectomy was performed and sent to pathology (Figure 4). Histologically, the fibrous and muscular tissue contained multiple epithelioid and multinucleated giant cell granulomas of varying size, composed of epithelioid cells and giant cells, surrounded by a lymphocytic rim and centered by caseous necrosis, consistent with tuberculosis (Figure 5). Following diagnosis, the patient received isoniazid, rifampin, and pyrazinamide for two months, then continued on isoniazid and rifampin to complete a total of nine months of therapy. At the two-year follow-up, there was no evidence of local recurrence, and the patient had resumed all normal daily activities.

## Discussion

Extrapulmonary tuberculosis is often identified late because of limited clinical suspicion. Skeletal involvement occurs in about 1%-5.2% of cases. Within this group, arthritis and spondylitis are the predominant forms, while bursitis and tenosynovitis are uncommon. Tuberculous trochanteric bursitis is infrequent and accounts for less than 2% of musculoskeletal tuberculosis [5]. In developed nations, bone and joint tuberculosis make up around 2.2-4.7% of all tuberculosis cases, with approximately 10-15% falling into the extrapulmonary category [6].

The clinical presentation of tuberculous trochanteric bursitis often lacks distinctive features and precision, requiring differentiation from tumors and other infections. It is noteworthy that fever or general symptoms are often absent in patients with tuberculous trochanteric bursitis, which accounts for the delay in diagnosis. Manifestations of musculoskeletal tuberculosis may be progressive over a long period, as the diagnosis remains uncertain and postponed [7]. 

Recurrence of tuberculosis has been documented in the literature, sometimes occurring several decades after completion of antituberculous treatment, and tuberculous bursitis can be a sign of reactivated disease [8]. Tuberculous coxitis is considered to develop secondary to previous pulmonary tuberculosis, resulting from hematogenous dissemination of Mycobacteria, and in a similar manner, tuberculous trochanteric bursitis can develop either via hematogenous transmission from a remote infectious focus or by direct spread from an adjacent site [9]. The occurrence of primary tuberculous trochanteritis in the absence of any other infectious focus or past history of tuberculosis renders our case particularly unusual and clinically noteworthy.

All imaging modalities can help suggest the diagnosis even when initial cultures are negative. MRI is particularly valuable in early diagnosis and is the imaging method of choice [10]. However, what makes our case particularly noteworthy is that MRI findings were misleading, being initially suggestive of hydatidosis due to the presence of multiple enhancing parietal villosities.

Confirming extrapulmonary tuberculosis requires obtaining samples from the affected sites for microscopy and culture, though the diagnostic yield of these methods remains limited. The final diagnosis is based on bacteriological or histological evidence, that is, culture of tubercle bacillus, synovial, and bone biopsy.

Clinical management differs greatly and recurrence is common. Several reports indicate a significant risk of recurrence in patients treated exclusively with antituberculous drugs, suggesting that surgical intervention should be considered as part of management. Although tuberculosis drugs can cure the disease at any stage, in some cases, treatment with tuberculosis drugs alone may result in recurrence. Therefore, surgery should be recommended in all patients with tuberculous trochanteric bursitis [11].

## Conclusions

Tuberculous trochanteric bursitis remains an exceptionally rare presentation of musculoskeletal tuberculosis. Manifestations usually occur insidiously, which delays diagnosis and treatment. Diagnosis is frequently delayed and often made only when abscesses or fistulas become evident. The high recurrence rate described in some series highlights the need to include surgery as part of the management strategy for all patients. Our case underlines the relevance of considering tuberculosis among the possible differential diagnoses of atypical soft tissue masses, even in the absence of systemic symptoms or previous contact history. The misleading MRI features, highly suggestive of hydatidosis, highlight the diagnostic challenges clinicians may face. Early suspicion, appropriate imaging, and histopathological confirmation are essential to avoid misdiagnosis and ensure timely management. Surgical treatment combined with anti-tubercular therapy provides favorable outcomes and should be considered in such unusual presentations.
